# Case report: Infectious mononucleosis with bilateral retinal haemorrhages under myelin oligodendrocyte glycoprotein antibody-associated disease

**DOI:** 10.3389/fimmu.2024.1480134

**Published:** 2024-11-06

**Authors:** Yuyu Li, Mingming Sun, Shihui Wei, Quangang Xu, Huanfen Zhou

**Affiliations:** Senior Department of Ophthalmology, the Third Medical Center of Chinese People’s Liberation Army (PLA) General Hospital & Chinese PLA Medical School, Beijing, China

**Keywords:** myelin-oligodendrocyte glycoprotein antibody-associated disease, optic neuritis, retinal haemorrhages, Epstein-Barr virus, infectious mononucleosis

## Abstract

**Background:**

Bilateral optic neuritis associated with optic disc swelling is a common feature of myelin oligodendrocyte glycoprotein antibody-associated disease (MOGAD). However, extensive deep retinal haemorrhages have not been described in the context of MOG-associated optic neuritis. Here, we report a case of infectious mononucleosis with marked binocular peripapillary and perivascular haemorrhages as well as extensive deep retinal haemorrhages in the presence of MOGAD.

**Case report:**

A 39-year-old Chinese woman presenting with subacute binocular vision reduction with no light perception was diagnosed with MOGAD. Fundus examination revealed the presence of binocular peripapillary and perivascular haemorrhages as well as extensive deep retinal haemorrhages with severe optic disc swelling greater in the right eye than in the left and dilated and tortuous retinal venules. The patient tested positive for the Epstein–Barr virus (EBV) antigen (595 U/mL) and the EBV capsid antigen (>750 U/mL). She had a fever and right upper quadrant abdominal pain, and a doctor determined splenomegaly 1 week before the onset of orbital pain and decreased vision acuity. Medical history and laboratory tests indicated the presence of concurrent infectious mononucleosis. Other investigational indicators of retinal haemorrhages, including hypertension, diabetes mellitus, vascular disease, systemic lupus erythematosus, metabolic disease, and renal or liver dysfunction, were absent.

**Discussion:**

This case suggests that retinal haemorrhage is a possible complication of infectious mononucleosis in the presence of MOGAD.

## Introduction

Myelin oligodendrocyte glycoprotein antibody-associated disease (MOGAD) is a recently identified autoimmune disorder characterised by inflammatory demyelination of the central nervous system in children or adults. Clinical features of MOGAD include unilateral or bilateral optic neuritis, transverse myelitis, acute disseminated encephalomyelitis, and brainstem demyelination (1). As much as 50%–70% of adult MOGAD patients present with optic neuritis at onset (2) with simultaneous bilateral involvement in up to 40%, average high-contrast visual acuity at nadir counting figures, optic nerve head swelling, and the possibility of peripapillary haemorrhage. Here, we report the case of a patient diagnosed with MOGAD who presented with binocular peripapillary and perivascular haemorrhages as well as extensive deep retinal haemorrhages similar to those seen in central retinal vein occlusion. Medical history and laboratory tests indicated the presence of concurrent infectious mononucleosis.

## Case report

**Figure 1:**
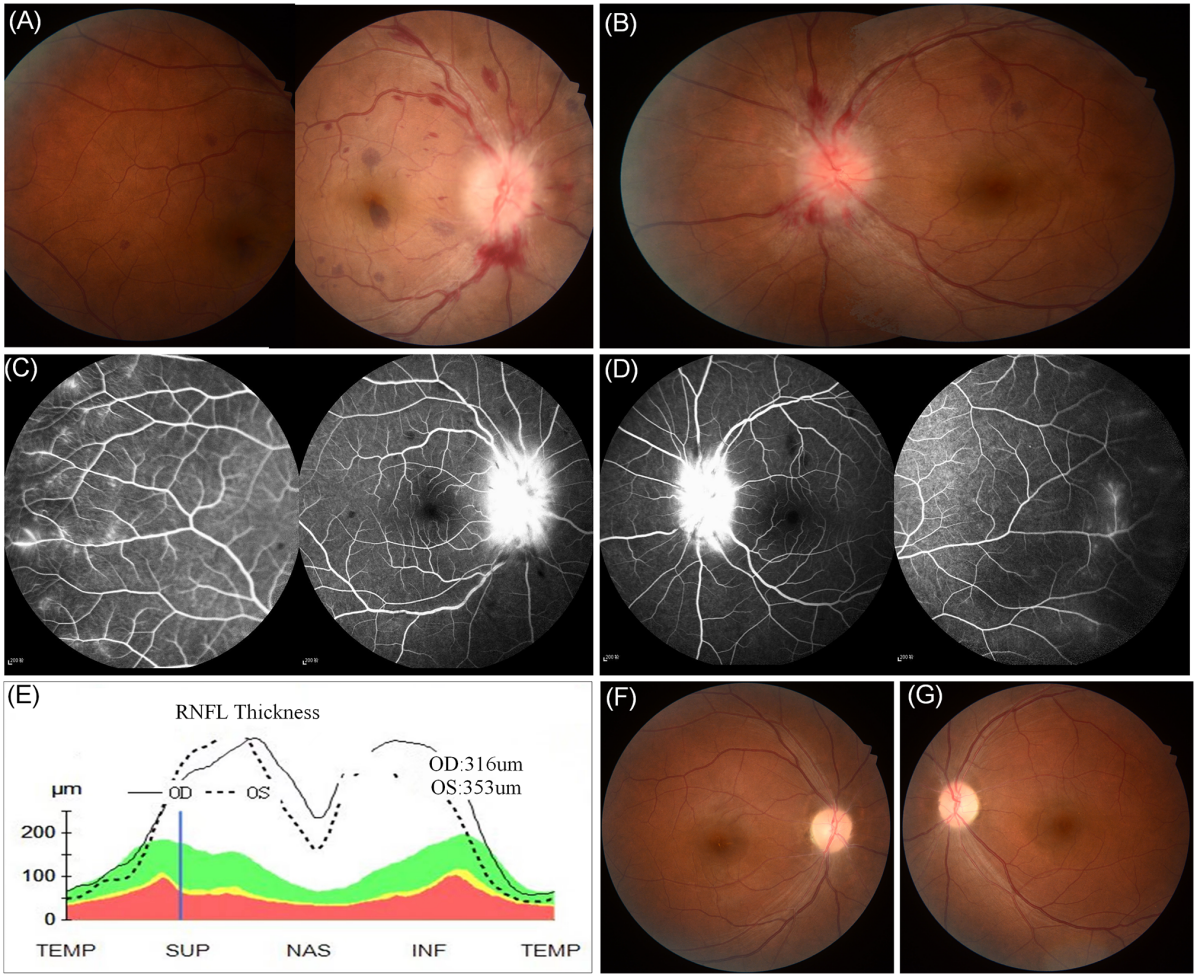
Fundus examination revealed the presence of binocular peripapillary and perivascular haemorrhages as well as extensive deep retinal haemorrhages with severe optic disc swelling greater in the right eye **(A)** than in the left **(B)**. **(C, D)** Fluorescein angiography images showed leakages of the bilateral optic disc and peripheral capillaries. **(E)** Retinal nerve fiber layer thickening on optical coherence tomography. **(F, G)** Retinal photos 1 month after onset showing the retinal haemorrhages in the binocular region had been absorbed completely.

**Figure 2:**
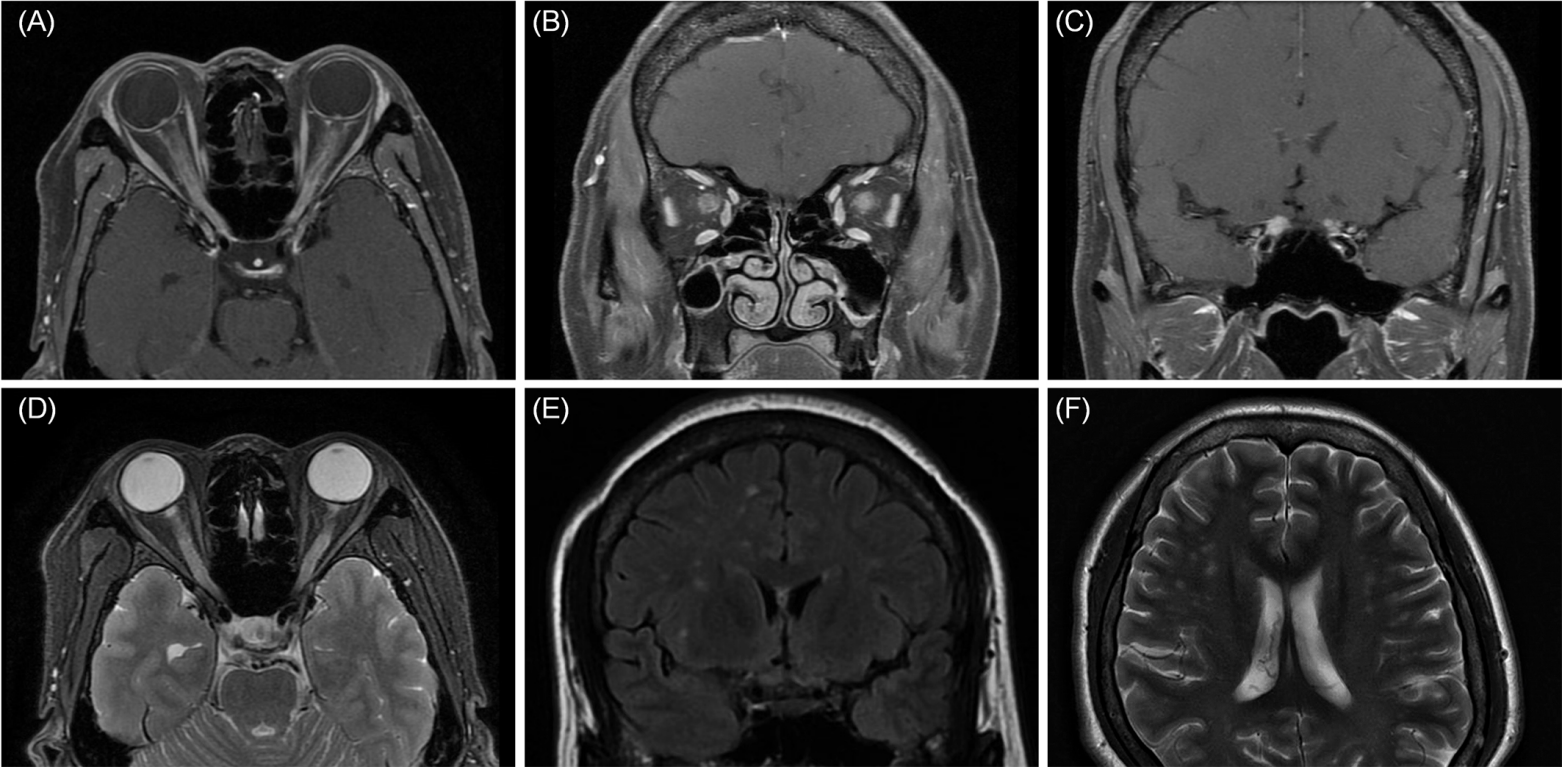
MRI axial **(A)** and coronal **(B, C)** showed bilateral enhancement of the optic nerve sheath, optic nerve, and chiasm. **(D)** Fat-suppressed T2-weighted sequences showed a hypersignal of the bilateral optic nerves and sheath. **(E, F)** Coronal and axial MRI was found to have multifocal patchy lesions in white matter of frontal cortex and the periventricular white matter.

On April 18, 2022, a 39-year-old Chinese woman complained of subacute binocular vision reduction for 4 days and no light perception for 2 days. She reported having a fever and right upper quadrant abdominal pain, and a doctor determined splenomegaly 25 days prior. The highest temperature recorded was 38.5°C, and the fever diminished after symptomatic treatment in an outside facility; no cause was identified. One week after treatment of the fever, the patient’s orbital pain worsened on eye movement, and she experienced a headache that lasted for 15 days. Four days before coming to our clinic, she presented with a subacute bilateral decrease to 20/250 in both eyes. At our clinic 2 days later, both eyes showed no light perception. The patient denied any toxic substance exposure, trauma, autoimmune disease, or family history of neuropathy. Examination showed that both pupils were fixed and dilated, with diameters of 7.5 mm and 6.5 mm in the right and left eyes, respectively. Fundus examination revealed binocular peripapillary, perivascular haemorrhages; extensive deep retinal haemorrhages with severe optic disc swelling, with right eye greater than left; and dilated and tortuous retinal venules. Fluorescein angiography images showed leakages of the bilateral optic disc and peripheral capillaries. Optical coherence tomography showed bilateral optic disc swelling (**Figures 1A–E**). Magnetic resonance imaging (MRI) showed bilateral enhancement of the optic nerve sheath, optic nerve, and chiasm on the axial and coronal sides. Fat-suppressed T2-weighted sequences showed a hypersignal of the bilateral optic nerves and sheath. T2 imaging revealed asymmetric multifocal patches of a bright signal that involved the left and right frontal cortices and the periventricular white matter of both parietal lobes (**Figures 2A–F**). The brainstem and spinal cord were normal. The patient tested positive for the Epstein–Barr virus (EBV) antigen (595 U/mL) and the EBV capsid antigen (>750 U/mL). The patient’s IL-6 serum was elevated (84.9 pg/mL), and the number of monocytes was normal. Tests for white blood cell count, platelet count, calcium, liver function, erythrocyte sedimentation rate, C-reactive protein, serum IgG4, anti-Ro, anti-La, rheumatoid factor, antinuclear antibody titre, and thyroxine were normal. While intracranial pressure was normal (120 mmH_2_O), cerebrospinal fluid analysis revealed a mildly elevated protein of 414.6 mg/L and a normal white blood cell count of 6 × 10^6^/L. The cerebrospinal fluid oligoclonal band was negative. A flow cytometric assay, which examines the biomarkers of central nervous system demyelinating diseases using live cell-based methods, showed that the MOG antibody was seropositive at a titre of 1:320, while the aquaporin 4 (AQP4) antibody was seronegative.

The MOGAD diagnostic criteria used in this case were based on the criteria proposed in 2023 (3). Multiple sclerosis was ruled out. Based on the presence of MOG-IgG, the typical clinical characteristics of optic neuritis, and MRI scans showing features common to patients with MOGAD, the diagnosis of MOGAD was definitive. Other indicators of retinal haemorrhages, including hypertension, diabetes mellitus, vascular disease, systemic lupus erythematosus, metabolic disease, and renal or liver dysfunction, were absent.

Because her visual function was severely impaired, the patient was treated with intravenous methylprednisolone (1,000 mg for 6 days) and plasma exchange, followed by oral prednisolone (1 mg/kg/day) with a slow taper over 3 months. By day 20 of the treatment, her pupils reduced in size to 5 mm and 4 mm in the right and left eyes, respectively, with visual acuity levels of 20/100 in the right eye and 20/40 in the left eye; the retinal haemorrhages in the binocular region had absorbed completely (**Figures 1F, G**). Due to the high risk of relapse in MOGAD patients (1), the patient was given 200 mg rituximab two times at an interval of 2 weeks. At the 3-month follow-up, her visual acuity had returned to normal (20/20 in both eyes). At the 12-month follow-up, the patient’s B cells were at 1%, so she was given an additional 200 mg of rituximab, and no relapse occurred.

## Discussion

The neuro-ophthalmic features in the case presented here were more severe than in general cases (1): the patient’s binocular visual acuity had decreased to no light perception, both pupils were fixed and dilated, and superficial peripapillary haemorrhages and extensive deep-layer retinal haemorrhages were observed in both eyes.

Haemorrhages of the optic disc or surrounding retina are typically few, small, and infrequent in cases of optic neuritis (4). One reported case of exception involved a 17-year-old Hispanic male individual who presented with unilateral dense premacular haemorrhages in the right eye associated with MOG optic neuritis (5). In contrast, the haemorrhages observed in our case were bilateral, superficial peripapillary haemorrhages accompanied by extensive deep-layer retinal haemorrhages. In addition, two case studies reported on paediatric patients who exhibited retinal haemorrhage in the context of acute disseminated encephalomyelitis associated with optic neuritis, but in those cases, MOG antibodies were negative and unknown (6, 7). Although the diagnosis in those cases differed from ours, acute disseminated encephalomyelitis associated with optic neuritis has a clinical manifestation similar to that of MOGAD.

In this case, the patient was positive for the EBV antigen, and the fever, right upper quadrant abdominal pain, and splenomegaly she experienced 1 week before the onset of orbital pain eased after symptomatic treatment. Infectious mononucleosis was confirmed based on the patient’s medical history and a positive test for the EBV antigen.

There are no studies about EBV and MOGAD, but studies have shown that chronic or recurrent EBV infection of the epithelial cells has been linked to systemic lupus erythematosus and Sjögren’s syndrome, whereas chronic/recurrent infection of B cells has been associated with rheumatoid arthritis, multiple sclerosis, and other diseases (8). Moreover, recent research found that EBV infection could trigger multiple sclerosis (9). MOGAD is thought to have an underlying autoimmune, demyelinating aetiology. It resembles multiple sclerosis. We can assume that maybe EBV infection triggers MOGAD in this case.

In a case of a patient diagnosed with infectious mononucleosis who complained of eye pain and blurred vision, the patient was found to have several retinal white-centred haemorrhages after 2 weeks of flu-like symptoms, including fever and sore throat; the patient had fully recovered at the last follow-up appointment (10). Our findings in this case are in line with this case report; it is likely that the retinal haemorrhage was caused by the infectious mononucleosis.

This case suggests the concurrent presence of MOGAD and infectious mononucleosis. Whether the retinal haemorrhage was due to the infectious mononucleosis or if the infectious mononucleosis was a causal agent for MOGAD in this case requires further consideration. Other factors associated with retinal haemorrhages, such as coagulation disorders, hypertension, hyperviscosity, leukaemia, and retinal dysplasia or telangiectasia, were absent. It is more likely that the retinal haemorrhage was caused by the infectious mononucleosis, with MOGAD being a coincidental complication. In this case, with the remission of the inflammation, the haemorrhage was completely absorbed, and the patient’s visual function returned to normal.

## Data Availability

The datasets presented in this article are not readily available because of ethical and privacy restrictions. Requests to access the datasets should be directed to the corresponding author.
